# Seroprevalence and Associated Risk Factors of Brucellosis among Indigenous Cattle in the Adamawa and North Regions of Cameroon

**DOI:** 10.1155/2018/3468596

**Published:** 2018-01-08

**Authors:** J. Awah-Ndukum, M. M. M. Mouiche, H. N. Bayang, V. Ngu Ngwa, E. Assana, K. J. M. Feussom, T. K. Manchang, P. A. Zoli

**Affiliations:** ^1^School of Veterinary Medicine and Sciences, University of Ngaoundéré, Ngaoundéré, Cameroon; ^2^Institute of Agricultural Research for Development, Veterinary Research Laboratory, Wakwa Regional Center, Ngaoundéré, Cameroon; ^3^Epidemio-Surveillance Service, Ministry of Livestock, Fisheries, Animal Industries, Yaoundé, Cameroon

## Abstract

A cross-sectional seroprevalence study was conducted on cattle in the North and Adamawa Regions of Cameroon to investigate the status of bovine brucellosis and identify potential risk factors. The diagnosis was carried out using the Rose Bengal Plate test (RBPT) and indirect ELISA (i-ELISA), while questionnaires were used to evaluate risk factors for bovine brucellosis in cattle. The Bayesian approach was used to evaluate the diagnostic tests' sensitivity and specificity. The overall individual level (*n* = 1031) and herd level (*n* = 82) seroprevalence were 5.4% (0.4–10.5) and 25.6% (16.2–35.0), respectively. Bayesian analysis revealed sensitivity of 58.3% (26.4–92.7) and 89.6% (80.4–99.4) and specificity of 92.1% (88.7–95.2) and 95.7% (91.1–99.7) for RBPT and i-ELISA, respectively. Management related factors such as region, locality, herd size, and knowledge of brucellosis and animal related factors such as sex and age were significantly associated with seropositivity of brucellosis. Zoonotic brucellosis is a neglected disease in Cameroon. The study highlights the need for control measures and the need to raise public awareness of the zoonotic occurrence and transmission of bovine brucellosis in the country. An integrated disease control strategy mimicking the one health approach involving medical personnel, veterinarians, related stakeholders, and affected communities cannot be overemphasized.

## 1. Introduction 

Brucellosis is an economically important and widespread zoonosis in the world caused by bacteria of the genus* Brucella, *which tend to infect specific animal species [1]. Brucellosis in cattle is usually caused by* B. abortus*, in sheep and goats by* B. melitensis*, and in swine by* B. suis* [2]. However, bovine brucellosis has occasionally been caused by* Brucella melitensis *and* Brucella suis* in some instances where mixed farming is practiced [2, 3], characterized by late term abortion, infertility, and reduced milk production [1, 2, 4]. Human brucellosis is mainly associated with* B. abortus*,* B. melitensis*, and* B. suis *[2]. Bovine brucellosis is widespread in Africa, where it remains one of the most important zoonotic diseases [5, 6], with prevalence ranging from 5% to over 70% in sub-Sahara African countries [1, 7, 8] including Cameroon [9–11]. Brucellosis has important public health significance but it is a “neglected zoonosis” in Cameroon. Poor implementation of essential control measures of zoonoses including animal brucellosis (e.g., restricting movement of infected cattle, reporting disease to the veterinary services, testing of animals) has been reported in the country [12]. There is little concerted veterinary and medical efforts to maximize zoonoses detection rates, while active involvement of the populations at risk and good health systems are also lacking.

The surveillance of bovine brucellosis in most countries in Africa including Cameroon is generally poor [6]. Lack of public awareness and poor or low income communities have been largely associated with the neglect of the disease [13]. The persistence and wide prevalence ranges of bovine brucellosis in sub-Sahara Africa are influenced by several factors associated with disease transmission between herds, factors influencing the maintenance and spread of infection within herds, purchase of infected cattle from livestock market for replacement or upgrading, nature of the animal production system, demographic factors, regulatory issues, climate, deforestation, and wildlife interaction [3, 7, 14, 15]. Although poorly implemented, the control of major zoonoses in Cameroon is mainly through the regulation of animal movement and postmortem examination of carcasses [12] such that appropriate preventive measures and planning of effective control programs cannot be achieved. Furthermore, vaccination of cattle against brucellosis is not practiced in Cameroon.

Serological diagnosis of brucellosis consists of testing sera by several tests, usually a screening test of high sensitivity, followed by a confirmatory test of high specificity [16]. However, presumptive seroprevalence studies in parts of Cameroon have shown that bovine brucellosis is endemic. For instance, using Rose Bengal Plate test (RBPT), complement fixation, indirect Enzyme-Linked Immunosorbent Assay (i-ELISA), and slow agglutination of Wright with ethylenediaminetetraacetic acid (EDTA) tests, seroprevalence rates in the range of 3–16% of brucellosis in cattle in parts of the Western Highlands and Adamawa Regions of Cameroon [9–11, 15, 17, 18] have been recorded. However, the performance and accuracy of a diagnostic test can be evaluated by comparing its sensitivity and specificity with those of a goal test or analyzing it with several tests using latent models [19–23]. The practice of transhumance (seasonal movement of people with their livestock from one pasture ground to another to improve grazing) among pastoralists, uncontrolled cross-boundary movements of animals, and mixing of herds during veterinary interventions are common and can facilitate the spread of the disease in Cameroon. The burden of brucellosis among various cattle populations and risk factors associated with the disease in different geographical parts of the country are not known.

Therefore, this study was carried out to evaluate diagnostic performance and accuracy of Rose Bengal Plate test (RBPT) and indirect Enzyme-Linked Immunosorbent Assay (i-ELISA) tests for screening and confirmation of bovine brucellosis using Bayesian method. The study also estimates the true seroprevalence of bovine brucellosis and assesses the potential risk factors for evidence-based disease control of the disease in Cameroon.

## 2. Materials and Methods

### 2.1. Description of Study Areas

**Figure 1:**
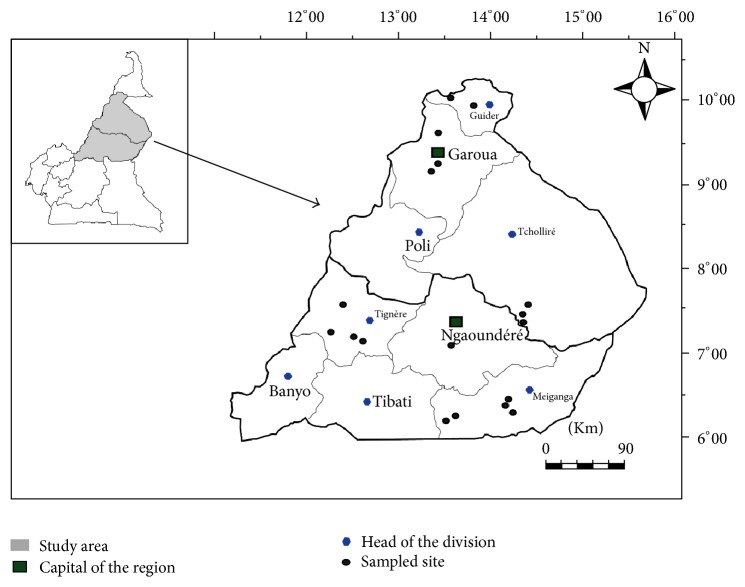
Map showing study areas in the North and Adamawa Regions of Cameroon.

The study was carried out in the two adjacent regions, the Adamawa and North Regions (Figure 1), of Cameroon (8°–13°N and 11°–16°E). The Adamawa Region is located in the Savannah Guinean Highland and the North Region in the Sudano-Sahelian, in the mid to high altitude zones of the country. Average annual precipitations of 1200–1600 mm, rainy season from about mid-March to October, temperature of 14°–26°C for the Adamawa Region and annual precipitations of 400–900 mm, four months of rainy season (July to October), temperature of 21°–36°C for the North Region have been noted. The Adamawa and North Regions are among the principal cattle production zones in Cameroon [24], and both regions were retained for the Dairy Development Program (Program to Improve Agricultural Productivity/Support for Development of the Dairy Sector (PAPA/ADFL)) of indigenous breeds (Guadali in Adamawa Region and Fulani/Bororo in North Region) to improve the availability of milk. The communities of the study areas are pure pastoralists (30%) and agropastoralists (65%) and practice predominantly the traditional systems of husbandry. The socioeconomic, political, cultural, and religious activities of the farmers are heavily dependent on cattle.* Bos indicus, Bos Taurus* (Namchi), and exotic (Montbeliarde, Holstein, Charolaise) breeds of cattle as well as their crossbreeds are reared in the study areas.

### 2.2. Selection of Study Animals

A cross-sectional study was carried out during the period of January to June 2014 using a stratified sampling procedure to select herds and then individual cattle per herd. A herd prevalence rate of 16%  [18] was used to estimate the sample sizes of herds as previously described [25]. Briefly, the selection of cattle herds was done by the random-number generation method of cattle keeping communities, cattle owners, and locations of herds listed on the PAPA/ADFL program in the Adamawa and North Regions. The herd sizes ranged from 20 to 80 animals and the selection procedure took into consideration costs, season, road accessibility (including distance and time to trek to herds), and local cultural beliefs (such as being suspicious of unfamiliar events in their farms and around their animals, as well as associating visitors to animal farms with reproductive failures and poor yield and performance) because a farmer's willingness to participate was never guaranteed. Only herds with a minimum of 10 head of cattle that are ≥2 years old and had spent ≥1 year in the area were included in the study. Herds that cograzed were grouped together and considered as one. Eligible herds from each study region were numbered and the study herds chosen randomly without replacing the number. Selection of individual cattle to be sampled from each chosen herd was based on a systematic random sampling technique as described by Asgedom et al. [4]. Individual cattle sampling of at least ten head of cattle per herd with a 25% sampling fraction from herds with >40 cattle was done. However, where random sampling was not possible in a chosen herd, ten head of cattle or qualified animals (if less than ten) were selected from those present and blood samples taken. Information related to the location, husbandry practices, breed, sex, and age of the animal were noted. The ages and breeds of the animals were provided by the farmers or otherwise determined as described earlier [26–28]. Herd level data including herd structure, size, history of purchases of animals, and farm management practices were also recorded.

Rose Bengal Plate test (RBPT) and indirect Enzyme-Linked Immunosorbent Assay (i-ELISA) test were performed on a total of 1031 head of cattle from 82 herds (18 villages communities) owned by 52 farmers. The animals in this study were reared traditionally with or without transhumance, as well as in semi-intensive and extensive systems. Apart from procedural restraining manipulations for safety purposes and jugular venipuncture for blood sampling (≥5 ml) using sterile vacutainer, the animals were not subjected to suffering. Serum samples were extracted from collected blood and stored at –20°C until laboratory analysis at the Veterinary Research Laboratory of IRAD, Wakwa Regional Center, Ngaoundéré, Cameroon.

### 2.3. Rose Bengal Plate Test

RBPT was performed as described by Alton et al. [29]. Briefly, the sera and antigen were brought to room temperature before use. Equal volumes (30 *μ*L) of standardized* B. abortus *antigen Weybridge strain 99 and test serum were mixed thoroughly and rotated on a glass plate using a stick applicator, and the plate was rocked for 4 min. The appearance of agglutination, recorded as positive, within 1 minute was scored 4+ (++++) and between 1 and 4 min was scored 1+ to 3+ (+, +  +, and +  +  +) according to the different degrees of agglutination. The absence of agglutination within 4 minutes was regarded as negative (−).

### 2.4. Indirect Enzyme-Linked Immunosorbent Assay

i-ELISA (ID.Vet®, Innovative Diagnostics, France) was performed according to the manufacturer's instructions and essentially as described by Limet et al. [30]. The test was conducted in 96-well polystyrene plate that was precoated with purified Brucella* abortus *lipopolysaccharide (LPS) antigen. A multispecies horseradish peroxidase (HRP) was used as conjugate as described by Saegerman et al. [31]. The substrate solution (TMB + DMSO + H_2_O_2_) was added after washing to eliminate excess conjugate. The coloration of antigen-antibody conjugate-peroxidase complex formed depended on the quantity of anti-*Brucella *antibodies that was present in the specimen tested. Thus, in the presence of antibodies, a blue solution appeared which became yellow after addition of the Stop Solution, while in the absence of antibodies, no coloration appeared. The microplate was read at 450 nm by an automatic ELISA reader and for each sample *S*/*P*% was calculated as follows:

(1) $$\begin{array}{c} \frac{S}{P}\%=\frac{\left({OD}_{sample}-{OD}_{nc}\right)}{\left({OD}_{pc}-{OD}_{nc}\right)}\times 100, \end{array}$$

where OD_sample_, OD_nc_, and OD_pc_ are the readings of optical densities for the sample, negative control, and positive control, respectively. The samples were classified as positive if *S*/*P*% ≥ 120%, negative if *S*/*P*% ≤ 110%, and doubtful if 110% < *S*/*P*% < 120%. Also, the fact that OD_pc_ > 0.350 and OD_pc_/OD_nc_ > 3 indicated that the test was working properly.

### 2.5. Risk Factor Analysis

Information on risk factors for bovine brucellosis was obtained by examination of individual cattle and herds and questionnaire interview with cattle professionals/handlers whose cattle herds were used in this study. The questionnaires were structured to collect information on a range of variables including animal management and husbandry practices, demographic information, and awareness of zoonotic brucellosis.

Risk assessments of the project were performed by the researchers to avoid hazards to all persons and animals involved in the project. Ethical clearances were obtained from the required authorities in Cameroon (PAPA/ADFL Program Cameroon, MINEPIA delegations in Adamawa and North Regions, School of Veterinary Medicine and Sciences/University of Ngaoundéré) before carrying out the study. The purpose of the study was explained to the farmers with the assistance of local veterinarians, community leaders, and trusted intermediaries. A herd was tested and interview questionnaire survey done after informed consent was given by the owner.

### 2.6. Data Analysis

The Bayesian approach (the appendix in Supplementary Materials (available here)) was used to evaluate the diagnostic tests' sensitivity and specificity and estimate the true prevalence, both based on conditional dependence between the tests in the absence of a gold standard method [32]. The sensitivity and specificity of the two tests were evaluated by subjecting each sample to the two tests and the observed data of the two tests summarized in cross tabulation. Briefly, the Bayesian model for one population–two tests was modified and applied for the data using WinBUGS free software [33–35]. The true prevalence of the disease was estimated as described by Rogan and Gladen [36].

Logistic regression model was used to test the significance of the effect of different risk factors on individual and herd level seroprevalence with statistical significance set at *P* < 0.05.

## 3. Results

### 3.1. Individual and Herd Level Seroprevalence Rates of Bovine Brucellosis

**Figure 2:**
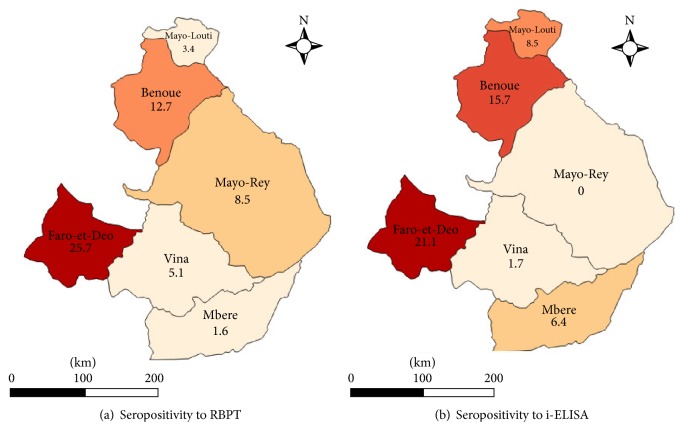
Map showing distribution of apparent seroprevalence rates at individual animal level according to locality and diagnostic test.

Combination of tests results of 1031 head of cattle revealed an overall apparent seroprevalence of 51 (5.0%  [3.7–6.3]) at individual animal level with 108 (10.8%  [8.6–12.3%]) for RBPT and 91 (8.8%  [7.1–10.5%]) for i-ELISA (Table 1). The seropositive reactors were 65 (12,2%  [9.4–15.0]) for RBPT and 60 (11,3%  [8,6; 14,0]) for i-ELISA in the Adamawa Region and 43 (8,2%  [5,8; 10,6]) for RBPT and 31 (6,1%  [4,0; 8,2]) for i-ELISA in the North Region. The distribution of seropositive reactors according to locality is shown in Figure 2.

From a total of 82 herds included in the study, 38 (46,2%  [35,4; 57,0]) herds for RBPT and 22 (26,6%  [17,0; 36,2]) herds for i-ELISA had at least one animal that tested positive. The herds were 20 (46,4%  [31,5; 61,3]) for RBPT and 15 (34,7%  [20,5; 48,9]) for i-ELISA in the Adamawa Region and 18 (46,0%  [30,3; 61,6]) for RBPT and 7 (17,7%  [5,7; 29,7]) for i-ELISA in the North Region.

A true prevalence of 5.4% (0.4–10.5) and test characteristics of 58.3% (26.4–92.7) and 89.6% (80.4–99.4) as sensitivity and 92.1% (88.7–95.2) and 95.7% (91.1–99.7) as specificity for RBPT and i-ELISA, respectively, were estimated after combining the results with expert's opinion on the Bayesian model using WinBUGS after 30,000 iterations.

### 3.2. Factors Affecting Seroprevalence of Bovine Brucellosis

The logistic regression revealed that region, locality, herd size, and knowledge of brucellosis as well as sex and age had effect (*P* < 0.05) on individual level seropositivity of brucellosis (Table 2). However, there was no difference (*P* > 0.05) between seroprevalence for cattle ≤ 4 years old (6.9% (2.3–11.5)) compared to cattle > 4 years old (9.1% (7.4–10.8)) (OR = 1.3 (0.6–2.8); *P* = 0.438). The seroprevalence at the individual cattle level was significantly higher in the Adamawa (11.5%) than in the North (6.1%) Region while Faro-et-Deo (21.1%), Benoue (15.7%), and Mayo-Louti (8.5%) localities and animals owned by farmers who were ignorant of brucellosis showed increased (*P* < 0.05) odds of having seropositive reactors than other localities and farmers who had knowledge of brucellosis, respectively. Also, animal seroprevalence significantly increased with the increase in herd size (Table 2). Breed, body condition, management system, contact with wildlife, abortion, stillbirth, and retained fetal membranes had no effects (*P* > 0.05) on the seroprevalence.

## 4. Discussion

The study revealed that bovine brucellosis is endemic in the Adamawa and North Regions of Cameroon and the overall individual level seroprevalence (10.5% for RBPT and 8.8% for i-ELISA) is different from those previously reported elsewhere in the country. Using competitive ELISA, Bayemi et al. [9] reported higher seroprevalence (8.4%) in Holstein cattle in the Northwest Region while Scolamacchia et al. [18] reported lower rates (3%) in indigenous cattle in Adamawa Region. Lower seroprevalence was also reported by Ojong [17] (4.6%) using RBPT in indigenous cattle in Northwest region. The finding of this study is close to the report of Shey-Njila et al. [11] (9.64%) who used indirect ELISA in indigenous cattle in the Western Highlands Regions. However, Bornarel and Akakpo [37] found a brucellosis seroprevalence of 12.5% in the Northern Cameroon and several other studies have reported brucellosis seroprevalence ranging from 7 to 31%  [37, 38, 39, 40, 41, 42]. Lower seroprevalence rates of brucellosis have been reported in indigenous cattle in Niger (1.3%) [38], Ivory Coast (4.6%) [34], Nigeria (3.9%) [3], Chad (2.6%) [1, 39], Central Africa Republic (3.3%) [40], Uganda (3.3%) [41], Zimbabwe (5.6%) [42, 43], and Ethiopia (2.4–3.9) [4, 7, 44]. Higher rates have been reported in Ivory Coast (8.8–10.3%) [35, 45], Zambia (18.7%) [46], Mali (22%) [47], Burkina Faso (13.2%) [48], and Algeria (9.7%) [49]. The herd level seroprevalence (46.3% (35.5–57.1) for RBPT and 30.5% (20.6–40.4) for i-ELISA) in the present study is higher than that reported by Scolamacchia et al. [18] (16%) in traditional extensive systems in Adamawa region. Similar herd prevalence was reported in Zimbabwe (25%) [42], while lower rates were reported in Ethiopia (9.2–15%) [7] and Niger (13.7%) [38] and higher rates in Ethiopia (42.31–45.9%) [4, 44, 50], Zimbabwe (40.0%) [42], and Algeria (31.5%) [49]. Relative sensitivity of 33% and specificity of 96% of delayed hypersensitivity test to brucellin (DHTB) in comparison to serological assays to detect brucellosis in zebu cattle were recorded in Northern Cameroon [51]. However, considering the limitations of DHTB and serological methods, the most sensitive brucellosis diagnostic procedure is a combination of both tests, where subjects are scored as positive if they are positive to either or both tests [51].

The relatively high levels of individual and herd seroprevalence recorded in this study are indications of high level introduction of infected animals to herds, transhumance, and high level mixing of herds such as grazing in communal pasture, livestock markets and, veterinary interventions. Variation in management practices (level of intensification and hygiene practices) [7, 14, 15, 43] in farms has been associated with differences in seroprevalence rates reports in various studies. However, the differences in prevalence rates reported in Cameroon and other parts of Africa could also be associated with the protocol adopted such as the type and number of diagnostic tests used. The protocol could have involved one test or more than one test in series (screening test followed by confirmation of positive reactors by another test) or in parallel (all tests are applied on the sampled animals independently). The evolution of the disease could be responsible for the different seroprevalence rates reported. Furthermore, close antigenic cross-reactivity with other bacterial infections* (Yersinia*,* Xanthomonas*,* Salmonella*,* Streptococci*,* E. coli, tuberculosis)* can lead to false positive results being encountered in serological diagnosis of brucellosis [35, 52, 53].

The* Brucella* ELISA test is generally considered to have higher sensitivity and specificity in determining* Brucella* specific antibodies than other serological tests [52, 54]. However, Cakan et al. [55] found that ELISA test for brucellosis was more sensitive only when both IgG and IgM were used, though their titre alone did not represent disease status. The sensitivity and specificity of ELISA IgG (45.6% and 97.1%, resp.) were reported to be lower than those of the standard tube agglutination test (95.6% and 100%, resp.) [55]. Therefore, results of both standard tube agglutination test and RBPT, which have similar sensitivity and specificity, should be interpreted according to the level of endemicity and seroprevalence rate of the population [53]. Estimation of sensitivity and specificity of a test requires knowledge of the true disease status and using a gold standard test. However, in the absence of such a gold test a Bayesian approach is used to evaluate the characteristics of the tests [20, 22, 23, 56]. Bayesian method provides a stable point and interval estimate without the necessity of large sample size [23]. The sensitivity of RBPT in the present study (58.3% (26.4–92.7%)) is similar to the finding of Sanogo et al. [34] (54.9% (23.5–95.1)) and lower than the finding of Getachew et al. [57] (89.6% (79.9–95.8)). The specificity of RBPT (92.1% (88.7–95.2) was fairly high and similar to the finding of Getachew et al. [57] (84.5% (68–94.8)) and Sanogo et al. [34] (97.7% (95.3–99.4)). The indirect ELISA in this study showed the best sensitivity (86.6% (80.4–99.4)) and specificity (95.7% (91.1–99.7)) for bovine brucellosis compared to RBPT and was similar to the finding of Sanogo et al. [34] who reported sensitivity (96.1% (92.7–99.8)) and specificity (95% (91.1–99.6)) and Gatechew et al. [57] who reported sensitivity (96.8% (92.3–99.1)) and specificity (96.3% (91.7–98.8)). This study estimated the true seroprevalence of brucellosis to be 5.4% (4–10.5) at individual animal level and 25.6% (16.2–35) at herd level. However, there are sporadic reports of outbreaks of bovine brucellosis in Cameroon to the World Organization For Animal Health, which is not indicative of the absence of the disease but rather of an underestimation [58], and other findings had concluded that the prevalence of the disease exceeds 5% in the country [59].

The higher seroprevalence recorded in the Adamawa Region (11.3%) compared to the North Region (6.1%) was associated with differences in climatic conditions between the regions. According to logistic regression analysis model, cattle in the Adamawa Region and areas where transhumance is practiced have increased odds of being seropositive than cattle in the North Region and areas which do not practice transhumance, respectively. The Adamawa Region has the typical tropical humid (Savannah Guinean) climate which is more favourable to the occurrence of brucellosis compared to the tropical dry (Sudano-Sahelian) climate of the North Region. This finding agrees with those of Sanogo et al. [34] who reported higher seroprevalence rates in Guinean zones than Sudano or Sudano-Sahelian zones in West Africa. Therefore, localities that practiced high transhumance and reduced levels of intensification revealed higher seroprevalence compared to more sedentary and intensified husbandry practices. Ibrahim et al. [7] and Boukary et al. [38] have reported that transhumance animals were major risk factor for brucellosis in sedentary animals and that the prevalence of brucellosis increased in sedentary herds that share same environments (pasture, water points, shelter) with animals on transhumance. In conformity with the seroprevalence rates reported in various parts of Africa [1, 3, 34, 35, 38, 40, 45, 46], previous works have observed higher rates in the more humid (4.6–9.6%) [9, 11, 17] than dryer (3%) [18] regions of Cameroon. However, the reason for higher individual seroprevalence rates recorded in regions with dry climates such as Mali [47], Burkina Faso [48], and Algeria [49] is not clear.

The study showed that female and old (≥9 years) cattle have increased odds of being seropositive reactors than bulls and younger cattle, respectively. This agrees with previous studies [3, 4, 15, 39, 45, 48] which explain that the economic and reproductive life of female cattle are much longer than those of male cattle and that the older the animal is, the longer the potential exposure to the disease is. Management practices in farms may play additional roles in the different seropositivity due to sex and age. However, Bayemi et al. [9] reported that young animals (≤3 years old) accounted for nearly half of the seropositive animals in small scale farms, while Akinseye et al. [3] and Ojong [17] did not observe differences in seropositivity due to sex.

Herd size was an important factor with significant effect on herd level and individual level seroprevalence, and the seroprevalence increased with herd size. This finding agrees with the reports of Berhe et al. [44], Boukary et al. [38], Makita et al. [8], and Sanogo et al. [34]. Though Asgedom et al. [4] did not observe difference in seropositivity with respect to herd size, an increase in herd size usually associated with poor hygiene of the farm [7] and stocking density has been reported as important determinant of brucellosis infection [60]. Furthermore, [43] have identified area, keeping mixed breed herds, stocking density, and herd size as independently associated with increased counts of seropositive cattle in a herd.

In addition to region, locality, and herd size, cattle owned by farmers who were ignorant of brucellosis showed more seropositivity than animals owned by farmers who had knowledge of brucellosis. Also, cattle owned by farmers who were ignorant of the occupational risk of brucellosis showed nonsignificant higher seropositivity than animals of farmers who had knowledge of the occupational risk of brucellosis. This could be explained by the fact that endemic zoonoses including bovine brucellosis remain widely neglected in low income countries [13] such as Cameroon, and there is lack of health and zoonoses education of farmers. Good hygiene practices [4] and protective effects towards animal and human brucellosis [61] in farms of owners with good knowledge of brucellosis have been reported. Poor hygiene practices and uncontrolled animals movements which posed high risks of transmitting brucellosis within and in between the herds were reported in farms where the owners were ignorant of brucellosis [4].

Breed, body condition, management system, contact with wildlife, abortion, stillbirth, and retained fetal membranes had no significant effects on the seroprevalence. The finding agrees with Asgedom et al. [4] regarding breed and with Ibrahim et al. [7] regarding management system but disagrees with Bayemi et al. [10] and Shirima et al. [62] who reported that animals on extensive management were more likely to be exposed to brucellosis compared to animals that are sedentary. This finding agrees with Kebede et al. [50] regarding abortion and retained fetal membranes and differs with Muma et al. [63], Ibrahim et al. [7], and Boukary et al. [38] with regard to the occurrence of abortion. However, the nonsignificant difference due to breed, management system, abortion, stillbirth, and retained fetal membranes recorded in this study was associated with high levels of uncontrolled movements and mixing of animals (transhumance and nontranshumance animals) irrespective of breed. Asgedom et al. [4] has revealed the existence of a strong association between number of services per conception and seropositivity of brucellosis. The number of services per conception increases when the cattle were repeatedly experiencing abortion, retained fetal membrane, and other reproductive health problems. Brucellosis affects the reproductive tract causing abortion and retained fetal membrane that usually leads to uterine infection and hence poor conception rate [1, 4]. The study agrees with Kungu et al. [64] for body condition in contrast to Bayemi et al. [10] who reported higher seroprevalence in animals with good body condition. Contrary to this study, brucellosis has been reported to be endemic in domestic animals in the livestock–wildlife interface areas [63], and contact with wildlife significantly increases seropositivity to the disease in domestic animals [15, 17]. Antibodies to* Brucella* spp. have been found in wildlife (e.g., buffalo) in Africa and cattle may become infected when in contact or sharing the same grazing area with infected animals of different species [15].

Brucellosis is endemic at different prevalence levels in parts of Cameroon. This study recorded overall moderate seroprevalence at individual animal level and high seroprevalence at herd level. Management related factors such as region, locality, herd size, and knowledge of brucellosis and animal related factors such as sex and age were associated with seropositivity of brucellosis. Brucellosis is a major public and animal health problem where bovine brucellosis is endemic. However, no specific control program exists at national level for zoonotic brucellosis in Cameroon. The need for an integrated disease control approach mimicking the one health approach and involving interdisciplinary strategies between animal and human health experts as well as concerned target stakeholders and affected communities cannot be overemphasized. This study provides important information on the epidemiology of bovine brucellosis in the Adamawa and North Regions of Cameroon and highlights the need for control measures and enhancing of public awareness of the zoonotic occurrence and transmission of bovine brucellosis in the country.

## Figures and Tables

**Table 1:** Combined results of Rose Bengal Plate test and indirect Enzyme-Linked Immunosorbent Assay among cattle in the North and Adamawa Regions of Cameroun (*n* = 1031).

Serological results	Number of cases (% [95% CI])
RBPT (+)	108 (10,5% [8,6; 12,4])
RBPT (−)	923 (89,5% [87,6; 91,4])
i-ELISA (+)	91 (8,8% [7,1; 10,5])
i-ELISA (−)	940 (91,2% [89,5; 92,9])
RBPT (+) i-ELISA (+)	51 (5,0% [3,7; 6,3])
RBPT (+) i-ELISA (−)	57 (5,5% [4,1; 6,9])
RBPT (−) i-ELISA (+)	40 (3,9% [2,7; 5,1])
RBPT (−) i-ELISA (−)	883 (85,6% [83,4; 87,7])
RBPT or i-ELISA (+)	148 (14,4% [12,2; 16,5])
RBPT or i-ELISA (−)	883 (85,6% [83,4; 87,7])

(−): negative; (+): positive; i-ELISA: indirect Enzyme-Linked Immunosorbent Assay; RBPT: Rose Bengal Plate test.

**Table 2:** Risk factor model for brucellosis seropositivity in individual cattle in Adamawa and North Regions of Cameroon (*n* = 1031).

Factor	Variable	Number^#^ (positive)	Seropositivity using i-ELISA [95% CI]	Odds ratio [95% CI]	*P* value
Region	North	509 (31)	6.1% [4.0–8.2]	1	-
	Adamawa	522 (60)	11.5% [8.8–14.2]	2.0 [1.2–3.1]	0.003

Locality	Vina	117 (2)	1.7% [0–4.0]	1	-
	Mbere	187 (12)	6.4% [2.9–9.9]	3.9 [0.9–17.9]	0.076
	Benoue	134 (21)	15.7% [9.5–21.8]	10.7 [2.4–46.6]	0.002
	Faro-et-Deo	218 (46)	21.1% [15.7–26.5]	15.4 [3.7–64.6]	<0.0001
	Mayo-Louti	117 (10)	8.5% [3.4–13.5]	5.4 [1.1–25.1]	0.032
	Mayo Rey	258 (0)	0%		

Herd size	≤30	69 (2)	2.9% [1.0–6.8]	1	-
	30–59	279 (38)	13.6% [9.6–17.6]	0.5 [0.3–0.8]	0.003
	≥60	683 (51)	7.5% [5.5–9.5]	0.2 [0.0–0.8]	0.024

Livestock systems	Semi-intensive	645 (55)	8.5% [6.3–10.6]	1	-
	Extensive	386 (36)	9.3% [6.4–12.2]	1.1 [0.7–1.7]	0.662

Contact with wildlife	Yes	476 (46)	9.7% [7.0–12.3]	1	-
	No	555 (45)	8.1% [5.8–10.4]	0.8 [0.5–1.3]	0.381

Knowledge of brucellosis	Yes	301 (15)	5.0% [2.5–7.5]	1	-
	No	339 (40)	11.8% [8.4–15.2]	2.5 [1.4–4.7]	0.003

Occupational risk of brucellosis	Yes	164 (10)	6.1% [2.4–9.8]	1	-
	No	476 (48)	10.1% [7.4–12.8]	1.7 [0.8–3.5]	0.129

Breed	Mbororo (red)	82 (5)	0.5% [0–2.0]	1	-
	Fulani (white)	167 (20)	1.9% [0–4.0]	1.5 [0.7–3.3]	0.295
	Gudali	715 (58)	5.6% [3.9–7.3]	1.5 [0.9–2.6]	0.123
	Crossbreed^*∗*^	67 (8)	0.8% [0–2.9]	0.6 [0.2–1.8]	0.374

Sex	Female	852 (83)	9.7% [7.7–11.7]	1	-
	Male	179 (8)	4.5% [1.5–7.5]	0.43 [0.20–0.91]	0.028

Age (years)	Young (≤4)	116 (8)	6.9% [2.3–11.5]	1	-
	Adult (5–8)	818 (65)	7.9% [6.0–9.7]	1.6 [0.9–2.8]	0.087
	Old (≥9)	97 (18)	18.6% [10.8–26.3]	3.8 [1.9–7.6]	<0.0001

Body Condition Score	Poor (<3)	33 (1)	3.0% [0.0–8.8]	1	-
	Good (3-4)	887 (82)	9% [7.3–11.1]	0.8 [0.3–1.6]	0.481
	Very Good (>4)	111 (8)	7.2% [2.4–12.0]	0.3 [0–2.3]	0.248

Abortion	Yes	310 (20)	6.5% [3.7–9.2]	1	-
	No	330 (35)	10.6% [7.3–13.9]	1.7 [1.0–3.0]	0.063

Stillbirth	Yes	244 (19)	7.8% [4.4–11.2]	1	-
	No	394 (36)	9.1% [6.3–11.9]	1.2 [0.7–2.1]	0.555

Retained placenta	Yes	162 (12)	8.0% [3.8–12.2]	1	-
	No	478 (42)	8.8% [6.3–11.3]	1.1 [0.6–2.1]	0.765

^#^Observed reactions of individual animals (*n* = 1031) or of animals of farmers who responded to questionnaire (*n* depends on number of animals of the farmer) were used in the category. ^*∗*^Crossbreed between local breeds.

## Supplementary Materials

Supplementary Materials Bayesian model for the estimation of the value of characteristics of the tests.
