# Supplementary material for: Seroprevalence and Associated Risk Factors of Brucellosis among Indigenous Cattle in the Adamawa and North Regions of Cameroon
Source: Vet Med Int. 2018 Jan 8;2018:3468596. doi: 10.1155/2018/3468596 (PMC5817279; doi:10.1155/2018/3468596)
Supplement: Supplementary Materials — Bayesian model for the estimation of the value of characteristics of the tests. [file 3468596.f1.docx]

**Supplementary Material: Bayesian model for the estimation of the value of characteristics of the tests**

Two by two contingency table for the Bayesian model :

| RBTT (test1) | I-ELISA (test 2) | Number |
| --- | --- | --- |
| + | + | 51 |
| + | - | 57 |
| - | + | 40 |
| - | - | 883 |

Bayesian model

model

{

result[1:4] ~ dmulti(pr[1:4], n)

pr[1] <- th[1]*th[2]*th[4]+(1-th[1])*(1-th[3])*(1-th[7])

pr[2] <- th[1]*th[2]*(1-th[4])+(1-th[1])*(1-th[3])*th[7]

pr[3] <- th[1]*(1-th[2])*th[5]+(1-th[1])*th[3]*(1-th[6])

pr[4] <- th[1]*(1-th[2])*(1-th[5])+(1-th[1])*th[3]*th[6]

th[1] ~ dbeta(1,1)

th[2] ~ dbeta(1,1)I(0.8,1)

th[3] ~ dbeta(1,1)I(0.9,1)

th[4] ~ dbeta(1,1)I(0.21,1)

th[5] ~ dbeta(1,1)

th[6] ~ dbeta(1,1)I(0.6,1)

th[7] ~ dbeta(1,1)

p<-th[1]

se[1]<-th[2]

sp[1]<-th[3]

se[2]<-th[2]*th[4]+(1-th[2])*th[5]

sp[2]<-th[3]*th[6]+(1-th[3])*th[7]

for (i in 1:4)

{

d[i] <- result[i]*log(max(result[i],1)/(pr[i]*n))

}

G0 <- 2 * sum(d[])

result2[1:4] ~ dmulti(pr[1:4], n)

for (i in 1:4)

{

d2[i] <- result2[i]*log(max(result2[i],1)/(pr[i]*n))

}

Gt<- 2 * sum(d2[])

bayesp<- step(G0 - Gt)

}

list(result=c(51,40,57,883), n=1031)
